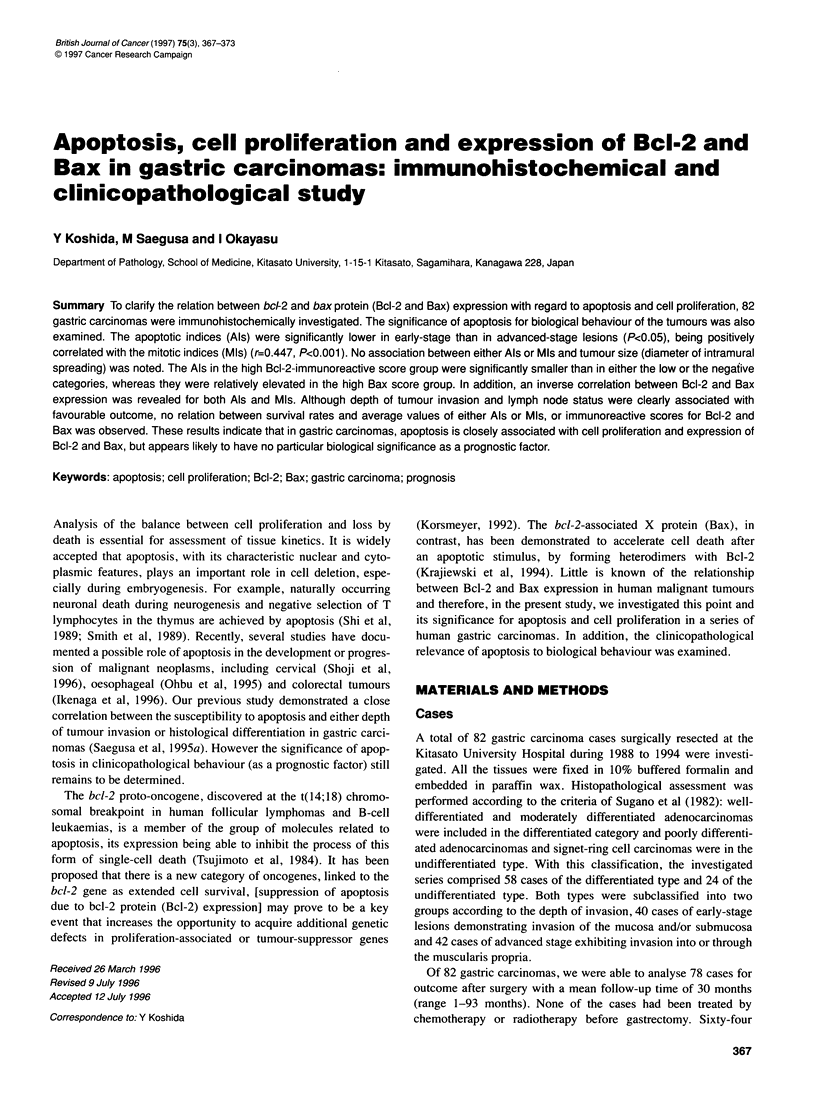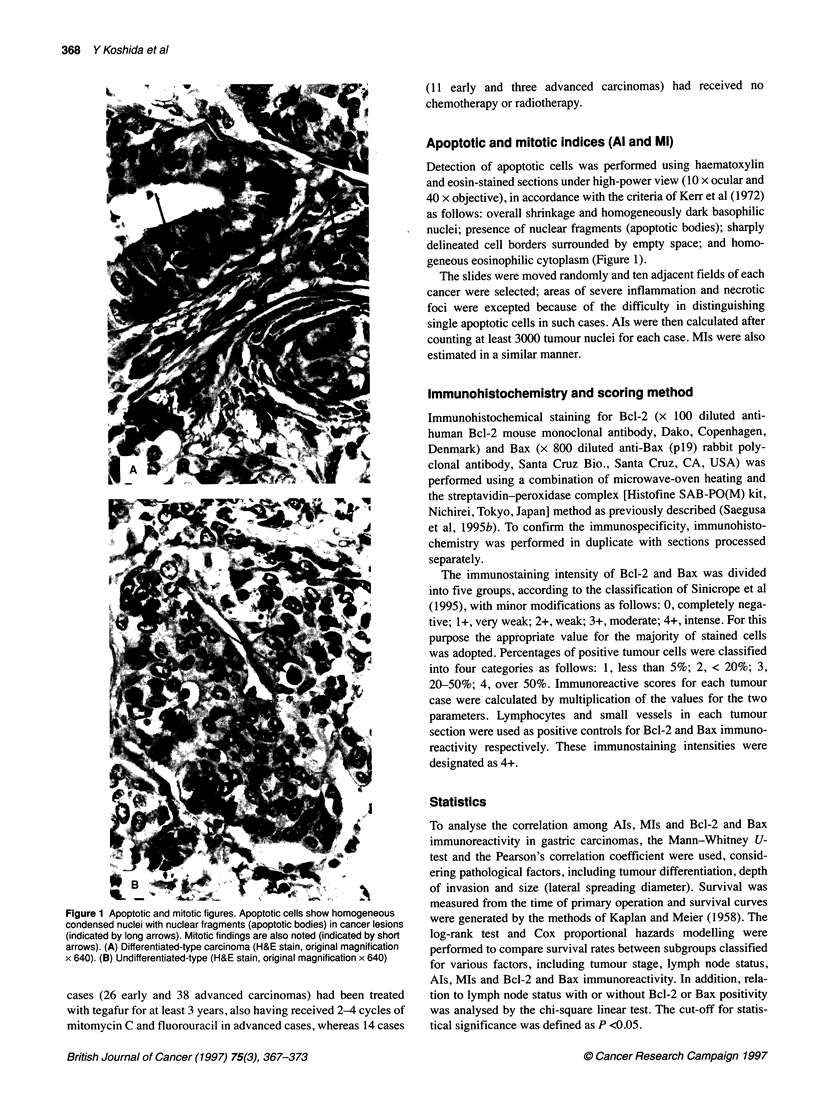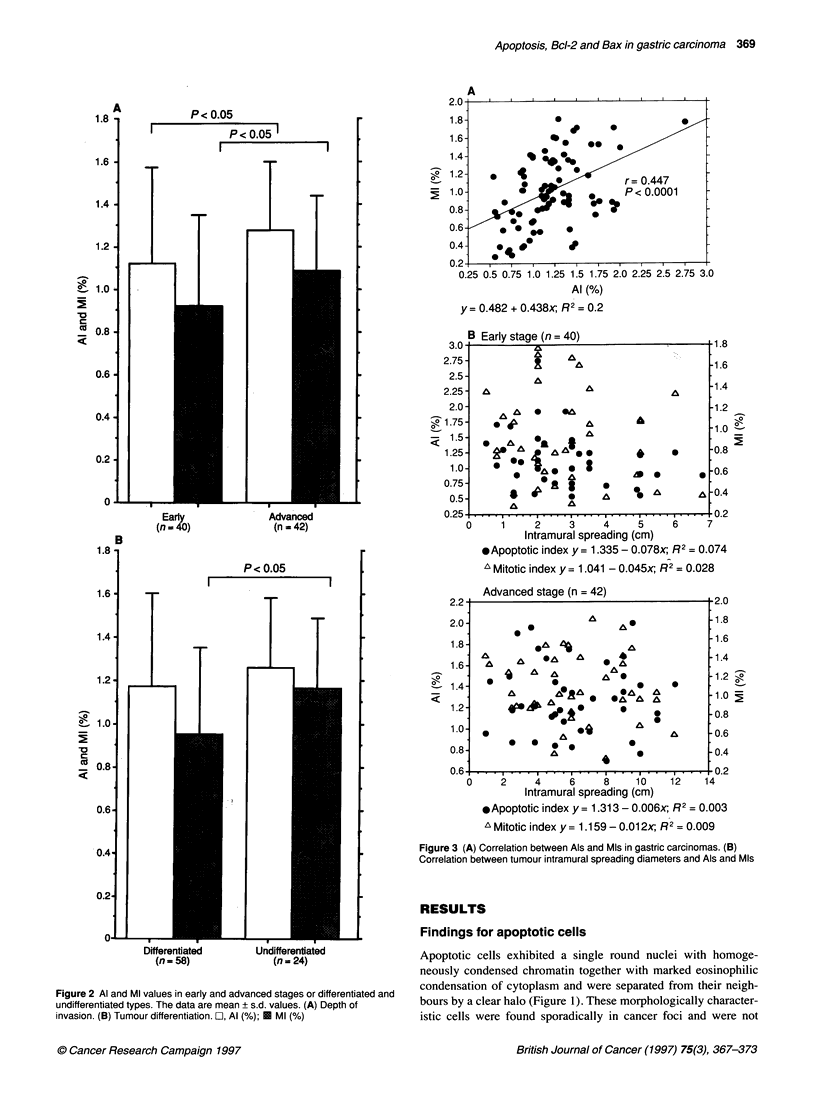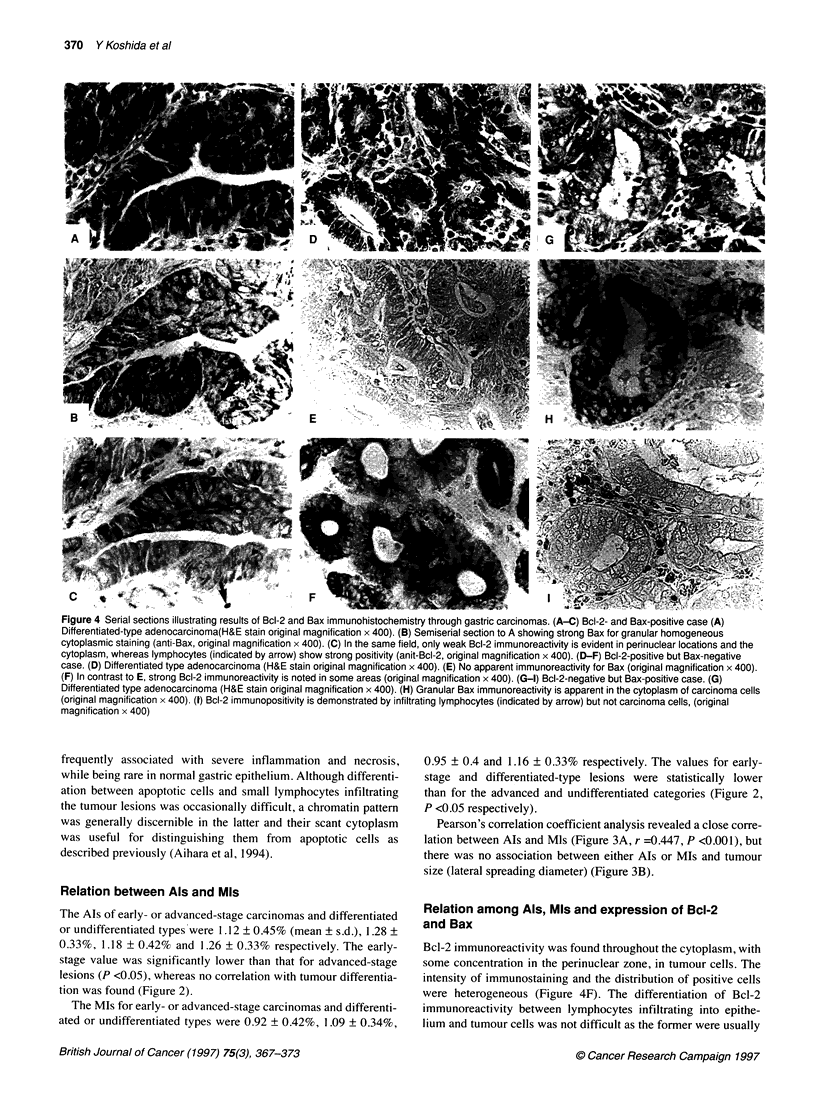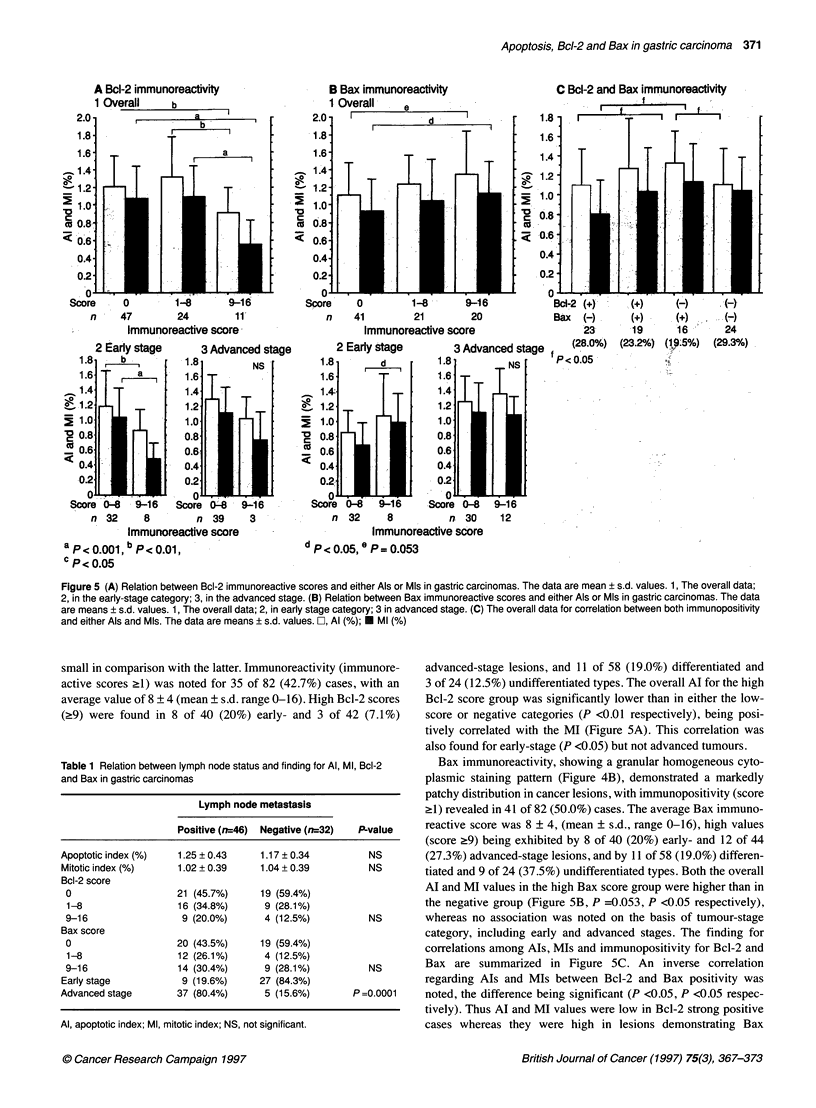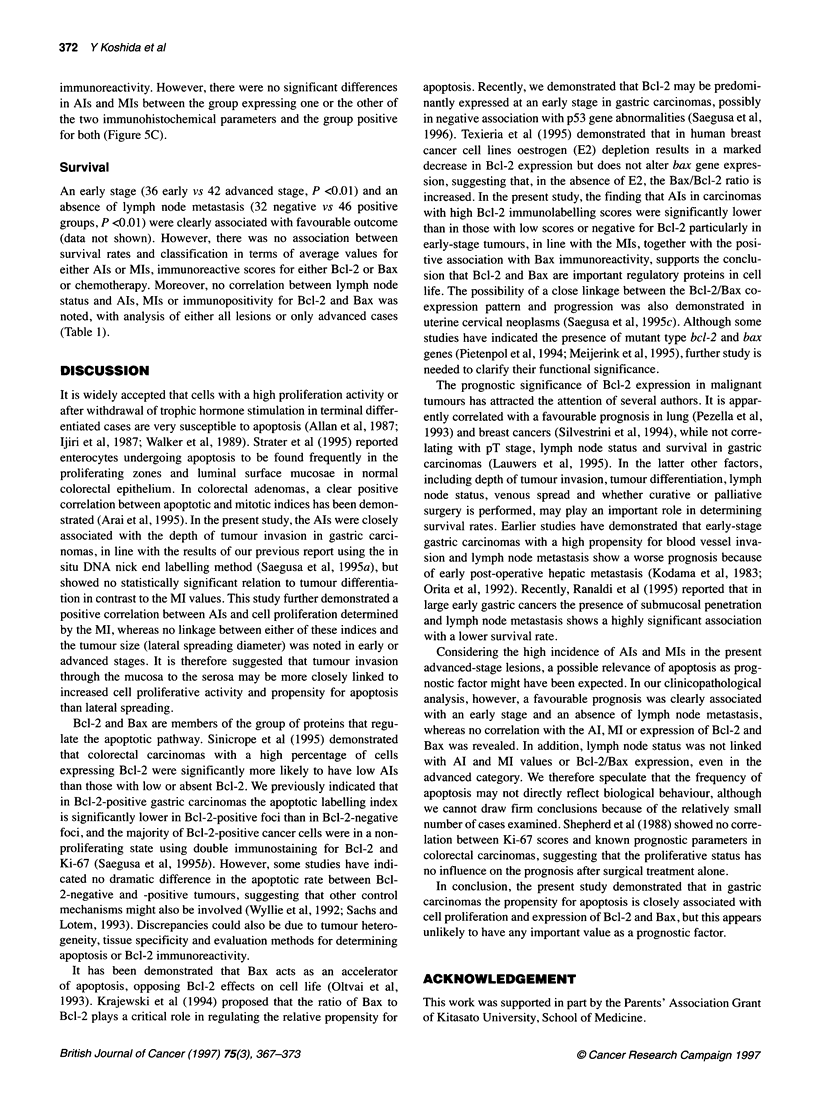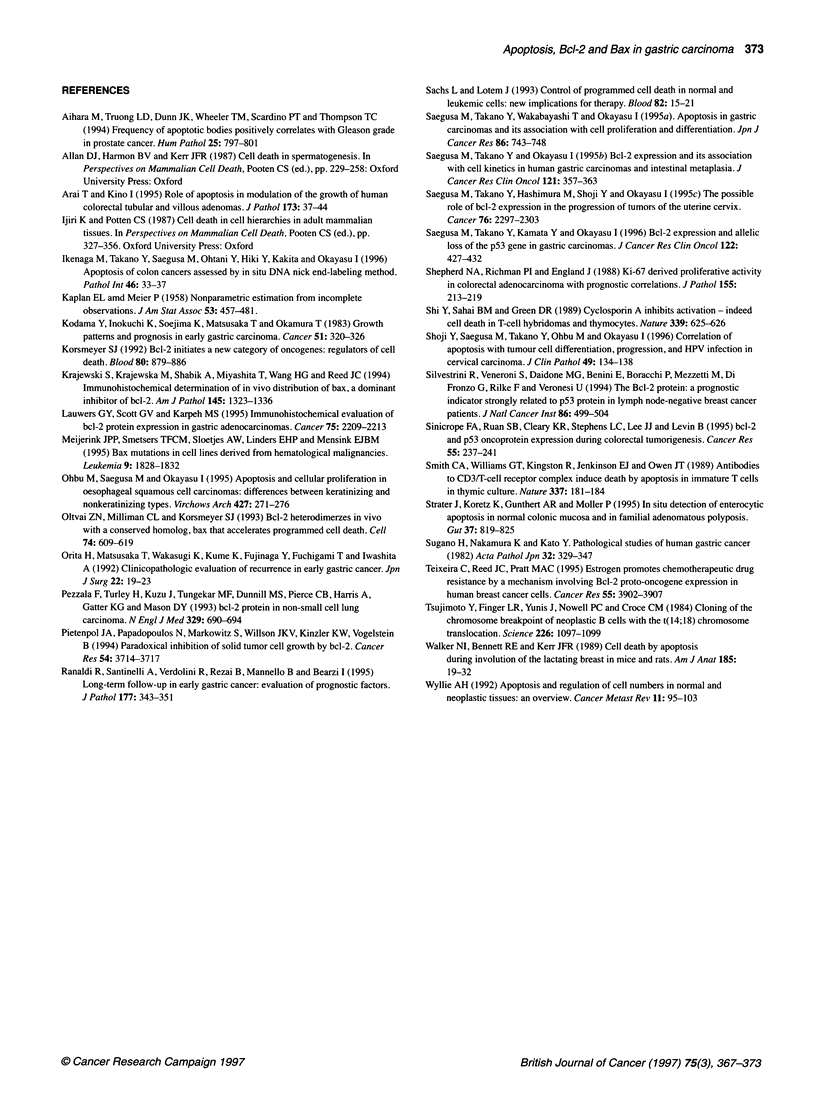# Apoptosis, cell proliferation and expression of Bcl-2 and Bax in gastric carcinomas: immunohistochemical and clinicopathological study.

**DOI:** 10.1038/bjc.1997.60

**Published:** 1997

**Authors:** Y. Koshida, M. Saegusa, I. Okayasu

**Affiliations:** Department of Pathology, School of Medicine, Kitasato University, Sagamihara, Kanagawa, Japan.

## Abstract

**Images:**


*British Journal of Cancer* (1997) **75**(3), 367–373
© 1997 Cancer Research Campaign

### Apoptosis, cell proliferation and expression of Bcl-2 and Bax in gastric carcinomas: immunohistochemical and clinicopathological study

**Y Koshida, M Saegusa and I Okayasu**

Department of Pathology, School of Medicine, Kitasato University, 1-15-1 Kitasato, Sagamihara, Kanagawa 228, Japan

**Summary** To clarify the relation between *bcl*-2 and *bax* protein (Bcl-2 and Bax) expression with regard to apoptosis and cell proliferation, 82 gastric carcinomas were immunohistochemically investigated. The significance of apoptosis for biological behaviour of the tumours was also examined. The apoptotic indices (AIs) were significantly lower in early-stage than in advanced-stage lesions ($P<0.05$), being positively correlated with the mitotic indices (MIs) ($r=0.447$, $P<0.001$). No association between either AIs or MIs and tumour size (diameter of intramural spreading) was noted. The AIs in the high Bcl-2-immunoreactive score group were significantly smaller than in either the low or the negative categories, whereas they were relatively elevated in the high Bax score group. In addition, an inverse correlation between Bcl-2 and Bax expression was revealed for both AIs and MIs. Although depth of tumour invasion and lymph node status were clearly associated with favourable outcome, no relation between survival rates and average values of either AIs or MIs, or immunoreactive scores for Bcl-2 and Bax was observed. These results indicate that in gastric carcinomas, apoptosis is closely associated with cell proliferation and expression of Bcl-2 and Bax, but appears likely to have no particular biological significance as a prognostic factor.

**Keywords**: apoptosis; cell proliferation; Bcl-2; Bax; gastric carcinoma; prognosis

Analysis of the balance between cell proliferation and loss by death is essential for assessment of tissue kinetics. It is widely accepted that apoptosis, with its characteristic nuclear and cytoplasmic features, plays an important role in cell deletion, especially during embryogenesis. For example, naturally occurring neuronal death during neurogenesis and negative selection of T lymphocytes in the thymus are achieved by apoptosis (Shi et al, 1989; Smith et al, 1989). Recently, several studies have documented a possible role of apoptosis in the development or progression of malignant neoplasms, including cervical (Shoji et al, 1996), oesophageal (Ohbu et al, 1995) and colorectal tumours (Ikenaga et al, 1996). Our previous study demonstrated a close correlation between the susceptibility to apoptosis and either depth of tumour invasion or histological differentiation in gastric carcinomas (Saegusa et al, 1995*a*). However the significance of apoptosis in clinicopathological behaviour (as a prognostic factor) still remains to be determined.

The *bcl-2* proto-oncogene, discovered at the t(14;18) chromosomal breakpoint in human follicular lymphomas and B-cell leukaemias, is a member of the group of molecules related to apoptosis, its expression being able to inhibit the process of this form of single-cell death (Tsujimoto et al, 1984). It has been proposed that there is a new category of oncogenes, linked to the *bcl-2* gene as extended cell survival, [suppression of apoptosis due to bcl-2 protein (Bcl-2) expression] may prove to be a key event that increases the opportunity to acquire additional genetic defects in proliferation-associated or tumour-suppressor genes (Korsmeyer, 1992). The *bcl-2*-associated X protein (Bax), in contrast, has been demonstrated to accelerate cell death after an apoptotic stimulus, by forming heterodimers with Bcl-2 (Krajiewski et al, 1994). Little is known of the relationship between Bcl-2 and Bax expression in human malignant tumours and therefore, in the present study, we investigated this point and its significance for apoptosis and cell proliferation in a series of human gastric carcinomas. In addition, the clinicopathological relevance of apoptosis to biological behaviour was examined.

### MATERIALS AND METHODS

### Cases

A total of 82 gastric carcinoma cases surgically resected at the Kitasato University Hospital during 1988 to 1994 were investigated. All the tissues were fixed in 10% buffered formalin and embedded in paraffin wax. Histopathological assessment was performed according to the criteria of Sugano et al (1982): well-differentiated and moderately differentiated adenocarcinomas were included in the differentiated category and poorly differentiated adenocarcinomas and signet-ring cell carcinomas were in the undifferentiated type. With this classification, the investigated series comprised 58 cases of the differentiated type and 24 of the undifferentiated type. Both types were subclassified into two groups according to the depth of invasion, 40 cases of early-stage lesions demonstrating invasion of the mucosa and/or submucosa and 42 cases of advanced stage exhibiting invasion into or through the muscularis propria.

Of 82 gastric carcinomas, we were able to analyse 78 cases for outcome after surgery with a mean follow-up time of 30 months (range 1–93 months). None of the cases had been treated by chemotherapy or radiotherapy before gastrectomy. Sixty-four

*Received 26 March 1996*
*Revised 9 July 1996*
*Accepted 12 July 1996*

*Correspondence to:* Y Koshida

**Figure 1** Apoptotic and mitotic figures. Apoptotic cells show homogeneous condensed nuclei with nuclear fragments (apoptotic bodies) in cancer lesions (indicated by long arrows). Mitotic findings are also noted (indicated by short arrows). (**A**) Differentiated-type carcinoma (H&E stain, original magnification × 640). (**B**) Undifferentiated-type (H&E stain, original magnification × 640)

cases (26 early and 38 advanced carcinomas) had been treated with tegafur for at least 3 years, also having received 2–4 cycles of mitomycin C and fluorouracil in advanced cases, whereas 14 cases (11 early and three advanced carcinomas) had received no chemotherapy or radiotherapy.

### Apoptotic and mitotic indices (AI and MI)

Detection of apoptotic cells was performed using haematoxylin and eosin-stained sections under high-power view (10 × ocular and 40 × objective), in accordance with the criteria of Kerr et al (1972) as follows: overall shrinkage and homogeneously dark basophilic nuclei; presence of nuclear fragments (apoptotic bodies); sharply delineated cell borders surrounded by empty space; and homogeneous eosinophilic cytoplasm (Figure 1).

The slides were moved randomly and ten adjacent fields of each cancer were selected; areas of severe inflammation and necrotic foci were excepted because of the difficulty in distinguishing single apoptotic cells in such cases. AIs were then calculated after counting at least 3000 tumour nuclei for each case. MIs were also estimated in a similar manner.

### Immunohistochemistry and scoring method

Immunohistochemical staining for Bcl-2 (× 100 diluted anti-human Bcl-2 mouse monoclonal antibody, Dako, Copenhagen, Denmark) and Bax (× 800 diluted anti-Bax (p19) rabbit polyclonal antibody, Santa Cruz Bio., Santa Cruz, CA, USA) was performed using a combination of microwave-oven heating and the streptavidin–peroxidase complex [Histofine SAB-PO(M) kit, Nichirei, Tokyo, Japan] method as previously described (Saegusa et al, 1995*b*). To confirm the immunospecificity, immunohistochemistry was performed in duplicate with sections processed separately.

The immunostaining intensity of Bcl-2 and Bax was divided into five groups, according to the classification of Sinicrope et al (1995), with minor modifications as follows: 0, completely negative; 1+, very weak; 2+, weak; 3+, moderate; 4+, intense. For this purpose the appropriate value for the majority of stained cells was adopted. Percentages of positive tumour cells were classified into four categories as follows: 1, less than 5%; 2, < 20%; 3, 20–50%; 4, over 50%. Immunoreactive scores for each tumour case were calculated by multiplication of the values for the two parameters. Lymphocytes and small vessels in each tumour section were used as positive controls for Bcl-2 and Bax immunoreactivity respectively. These immunostaining intensities were designated as 4+.

### Statistics

To analyse the correlation among AIs, MIs and Bcl-2 and Bax immunoreactivity in gastric carcinomas, the Mann–Whitney *U*-test and the Pearson's correlation coefficient were used, considering pathological factors, including tumour differentiation, depth of invasion and size (lateral spreading diameter). Survival was measured from the time of primary operation and survival curves were generated by the methods of Kaplan and Meier (1958). The log-rank test and Cox proportional hazards modelling were performed to compare survival rates between subgroups classified for various factors, including tumour stage, lymph node status, AIs, MIs and Bcl-2 and Bax immunoreactivity. In addition, relation to lymph node status with or without Bcl-2 or Bax positivity was analysed by the chi-square linear test. The cut-off for statistical significance was defined as $P < 0.05$.

© Cancer Research Campaign 1997

Figure 2 AI and MI values in early and advanced stages or differentiated and undifferentiated types. The data are mean ± s.d. values. (A) Depth of invasion. (B) Tumour differentiation. □, AI (%); ▩ MI (%)

Figure 3 (A) Correlation between AIs and MIs in gastric carcinomas. (B) Correlation between tumour intramural spreading diameters and AIs and MIs

### RESULTS

### Findings for apoptotic cells

Apoptotic cells exhibited a single round nuclei with homogeneously condensed chromatin together with marked eosinophilic condensation of cytoplasm and were separated from their neighbours by a clear halo (Figure 1). These morphologically characteristic cells were found sporadically in cancer foci and were not

**Figure 4** Serial sections illustrating results of Bcl-2 and Bax immunohistochemistry through gastric carcinomas. (**A–C**) Bcl-2- and Bax-positive case (**A**) Differentiated-type adenocarcinoma(H&E stain original magnification × 400). (**B**) Semiserial section to A showing strong Bax for granular homogeneous cytoplasmic staining (anti-Bax, original magnification × 400). (**C**) In the same field, only weak Bcl-2 immunoreactivity is evident in perinuclear locations and the cytoplasm, whereas lymphocytes (indicated by arrow) show strong positivity (anit-Bcl-2, original magnification × 400). (**D–F**) Bcl-2-positive but Bax-negative case. (**D**) Differentiated type adenocarcinoma (H&E stain original magnification × 400). (**E**) No apparent immunoreactivity for Bax (original magnification × 400). (**F**) In contrast to **E**, strong Bcl-2 immunoreactivity is noted in some areas (original magnification × 400). (**G–I**) Bcl-2-negative but Bax-positive case. (**G**) Differentiated type adenocarcinoma (H&E stain original magnification × 400). (**H**) Granular Bax immunoreactivity is apparent in the cytoplasm of carcinoma cells (original magnification × 400). (**I**) Bcl-2 immunopositivity is demonstrated by infiltrating lymphocytes (indicated by arrow) but not carcinoma cells, (original magnification × 400)

frequently associated with severe inflammation and necrosis, while being rare in normal gastric epithelium. Although differentiation between apoptotic cells and small lymphocytes infiltrating the tumour lesions was occasionally difficult, a chromatin pattern was generally discernible in the latter and their scant cytoplasm was useful for distinguishing them from apoptotic cells as described previously (Aihara et al, 1994).

### Relation between AIs and MIs

The AIs of early- or advanced-stage carcinomas and differentiated or undifferentiated types were 1.12 ± 0.45% (mean ± s.d.), 1.28 ± 0.33%, 1.18 ± 0.42% and 1.26 ± 0.33% respectively. The early-stage value was significantly lower than that for advanced-stage lesions ($P < 0.05$), whereas no correlation with tumour differentiation was found (Figure 2).

The MIs for early- or advanced-stage carcinomas and differentiated or undifferentiated types were 0.92 ± 0.42%, 1.09 ± 0.34%, 0.95 ± 0.4 and 1.16 ± 0.33% respectively. The values for early-stage and differentiated-type lesions were statistically lower than for the advanced and undifferentiated categories (Figure 2, $P < 0.05$ respectively).

Pearson's correlation coefficient analysis revealed a close correlation between AIs and MIs (Figure 3A, $r = 0.447$, $P < 0.001$), but there was no association between either AIs or MIs and tumour size (lateral spreading diameter) (Figure 3B).

### Relation among AIs, MIs and expression of Bcl-2 and Bax

Bcl-2 immunoreactivity was found throughout the cytoplasm, with some concentration in the perinuclear zone, in tumour cells. The intensity of immunostaining and the distribution of positive cells were heterogeneous (Figure 4F). The differentiation of Bcl-2 immunoreactivity between lymphocytes infiltrating into epithelium and tumour cells was not difficult as the former were usually

© Cancer Research Campaign 1997

**Figure 5** (**A**) Relation between Bcl-2 immunoreactive scores and either AIs or MIs in gastric carcinomas. The data are mean ± s.d. values. 1, The overall data; 2, in the early-stage category; 3, in the advanced stage. (**B**) Relation between Bax immunoreactive scores and either AIs or MIs in gastric carcinomas. The data are means ± s.d. values. 1, The overall data; 2, in early stage category; 3 in advanced stage. (**C**) The overall data for correlation between both immunopositivity and either AIs and MIs. The data are means ± s.d. values. □, AI (%); ■ MI (%)

small in comparison with the latter. Immunoreactivity (immunoreactive scores ≥1) was noted for 35 of 82 (42.7%) cases, with an average value of 8 ± 4 (mean ± s.d. range 0–16). High Bcl-2 scores (≥9) were found in 8 of 40 (20%) early- and 3 of 42 (7.1%) advanced-stage lesions, and 11 of 58 (19.0%) differentiated and 3 of 24 (12.5%) undifferentiated types. The overall AI for the high Bcl-2 score group was significantly lower than in either the low-score or negative categories ($P$ <0.01 respectively), being positively correlated with the MI (Figure 5A). This correlation was also found for early-stage ($P$ <0.05) but not advanced tumours.

**Table 1** Relation between lymph node status and finding for AI, MI, Bcl-2 and Bax in gastric carcinomas

| | Lymph node metastasis | | |
|---|---|---|---|
| | **Positive (*n*=46)** | **Negative (*n*=32)** | ***P*-value** |
| Apoptotic index (%) | 1.25 ± 0.43 | 1.17 ± 0.34 | NS |
| Mitotic index (%) | 1.02 ± 0.39 | 1.04 ± 0.39 | NS |
| Bcl-2 score | | | |
| 0 | 21 (45.7%) | 19 (59.4%) | |
| 1–8 | 16 (34.8%) | 9 (28.1%) | |
| 9–16 | 9 (20.0%) | 4 (12.5%) | NS |
| Bax score | | | |
| 0 | 20 (43.5%) | 19 (59.4%) | |
| 1–8 | 12 (26.1%) | 4 (12.5%) | |
| 9–16 | 14 (30.4%) | 9 (28.1%) | NS |
| Early stage | 9 (19.6%) | 27 (84.3%) | |
| Advanced stage | 37 (80.4%) | 5 (15.6%) | $P$ =0.0001 |

AI, apoptotic index; MI, mitotic index; NS, not significant.

Bax immunoreactivity, showing a granular homogeneous cytoplasmic staining pattern (Figure 4B), demonstrated a markedly patchy distribution in cancer lesions, with immunopositivity (score ≥1) revealed in 41 of 82 (50.0%) cases. The average Bax immunoreactive score was 8 ± 4, (mean ± s.d., range 0–16), high values (score ≥9) being exhibited by 8 of 40 (20%) early- and 12 of 44 (27.3%) advanced-stage lesions, and by 11 of 58 (19.0%) differentiated and 9 of 24 (37.5%) undifferentiated types. Both the overall AI and MI values in the high Bax score group were higher than in the negative group (Figure 5B, $P$ =0.053, $P$ <0.05 respectively), whereas no association was noted on the basis of tumour-stage category, including early and advanced stages. The finding for correlations among AIs, MIs and immunopositivity for Bcl-2 and Bax are summarized in Figure 5C. An inverse correlation regarding AIs and MIs between Bcl-2 and Bax positivity was noted, the difference being significant ($P$ <0.05, $P$ <0.05 respectively). Thus AI and MI values were low in Bcl-2 strong positive cases whereas they were high in lesions demonstrating Bax

© Cancer Research Campaign 1997

immunoreactivity. However, there were no significant differences in AIs and MIs between the group expressing one or the other of the two immunohistochemical parameters and the group positive for both (Figure 5C).

### Survival

An early stage (36 early *vs* 42 advanced stage, $P < 0.01$) and an absence of lymph node metastasis (32 negative *vs* 46 positive groups, $P < 0.01$) were clearly associated with favourable outcome (data not shown). However, there was no association between survival rates and classification in terms of average values for either AIs or MIs, immunoreactive scores for either Bcl-2 or Bax or chemotherapy. Moreover, no correlation between lymph node status and AIs, MIs or immunopositivity for Bcl-2 and Bax was noted, with analysis of either all lesions or only advanced cases (Table 1).

### DISCUSSION

It is widely accepted that cells with a high proliferation activity or after withdrawal of trophic hormone stimulation in terminal differentiated cases are very susceptible to apoptosis (Allan et al, 1987; Ijiri et al, 1987; Walker et al, 1989). Strater et al (1995) reported enterocytes undergoing apoptosis to be found frequently in the proliferating zones and luminal surface mucosae in normal colorectal epithelium. In colorectal adenomas, a clear positive correlation between apoptotic and mitotic indices has been demonstrated (Arai et al, 1995). In the present study, the AIs were closely associated with the depth of tumour invasion in gastric carcinomas, in line with the results of our previous report using the in situ DNA nick end labelling method (Saegusa et al, 1995*a*), but showed no statistically significant relation to tumour differentiation in contrast to the MI values. This study further demonstrated a positive correlation between AIs and cell proliferation determined by the MI, whereas no linkage between either of these indices and the tumour size (lateral spreading diameter) was noted in early or advanced stages. It is therefore suggested that tumour invasion through the mucosa to the serosa may be more closely linked to increased cell proliferative activity and propensity for apoptosis than lateral spreading.

Bcl-2 and Bax are members of the group of proteins that regulate the apoptotic pathway. Sinicrope et al (1995) demonstrated that colorectal carcinomas with a high percentage of cells expressing Bcl-2 were significantly more likely to have low AIs than those with low or absent Bcl-2. We previously indicated that in Bcl-2-positive gastric carcinomas the apoptotic labelling index is significantly lower in Bcl-2-positive foci than in Bcl-2-negative foci, and the majority of Bcl-2-positive cancer cells were in a non-proliferating state using double immunostaining for Bcl-2 and Ki-67 (Saegusa et al, 1995*b*). However, some studies have indicated no dramatic difference in the apoptotic rate between Bcl-2-negative and -positive tumours, suggesting that other control mechanisms might also be involved (Wyllie et al, 1992; Sachs and Lotem, 1993). Discrepancies could also be due to tumour heterogeneity, tissue specificity and evaluation methods for determining apoptosis or Bcl-2 immunoreactivity.

It has been demonstrated that Bax acts as an accelerator of apoptosis, opposing Bcl-2 effects on cell life (Oltvai et al, 1993). Krajewski et al (1994) proposed that the ratio of Bax to Bcl-2 plays a critical role in regulating the relative propensity for apoptosis. Recently, we demonstrated that Bcl-2 may be predominantly expressed at an early stage in gastric carcinomas, possibly in negative association with p53 gene abnormalities (Saegusa et al, 1996). Texieria et al (1995) demonstrated that in human breast cancer cell lines oestrogen (E2) depletion results in a marked decrease in Bcl-2 expression but does not alter *bax* gene expression, suggesting that, in the absence of E2, the Bax/Bcl-2 ratio is increased. In the present study, the finding that AIs in carcinomas with high Bcl-2 immunolabelling scores were significantly lower than in those with low scores or negative for Bcl-2 particularly in early-stage tumours, in line with the MIs, together with the positive association with Bax immunoreactivity, supports the conclusion that Bcl-2 and Bax are important regulatory proteins in cell life. The possibility of a close linkage between the Bcl-2/Bax co-expression pattern and progression was also demonstrated in uterine cervical neoplasms (Saegusa et al, 1995*c*). Although some studies have indicated the presence of mutant type *bcl-2* and *bax* genes (Pietenpol et al, 1994; Meijerink et al, 1995), further study is needed to clarify their functional significance.

The prognostic significance of Bcl-2 expression in malignant tumours has attracted the attention of several authors. It is apparently correlated with a favourable prognosis in lung (Pezella et al, 1993) and breast cancers (Silvestrini et al, 1994), while not correlating with pT stage, lymph node status and survival in gastric carcinomas (Lauwers et al, 1995). In the latter other factors, including depth of tumour invasion, tumour differentiation, lymph node status, venous spread and whether curative or palliative surgery is performed, may play an important role in determining survival rates. Earlier studies have demonstrated that early-stage gastric carcinomas with a high propensity for blood vessel invasion and lymph node metastasis show a worse prognosis because of early post-operative hepatic metastasis (Kodama et al, 1983; Orita et al, 1992). Recently, Ranaldi et al (1995) reported that in large early gastric cancers the presence of submucosal penetration and lymph node metastasis shows a highly significant association with a lower survival rate.

Considering the high incidence of AIs and MIs in the present advanced-stage lesions, a possible relevance of apoptosis as prognostic factor might have been expected. In our clinicopathological analysis, however, a favourable prognosis was clearly associated with an early stage and an absence of lymph node metastasis, whereas no correlation with the AI, MI or expression of Bcl-2 and Bax was revealed. In addition, lymph node status was not linked with AI and MI values or Bcl-2/Bax expression, even in the advanced category. We therefore speculate that the frequency of apoptosis may not directly reflect biological behaviour, although we cannot draw firm conclusions because of the relatively small number of cases examined. Shepherd et al (1988) showed no correlation between Ki-67 scores and known prognostic parameters in colorectal carcinomas, suggesting that the proliferative status has no influence on the prognosis after surgical treatment alone.

In conclusion, the present study demonstrated that in gastric carcinomas the propensity for apoptosis is closely associated with cell proliferation and expression of Bcl-2 and Bax, but this appears unlikely to have any important value as a prognostic factor.

### ACKNOWLEDGEMENT

This work was supported in part by the Parents' Association Grant of Kitasato University, School of Medicine.

© Cancer Research Campaign 1997

### REFERENCES

Aihara M, Truong LD, Dunn JK, Wheeler TM, Scardino PT and Thompson TC (1994) Frequency of apoptotic bodies positively correlates with Gleason grade in prostate cancer. *Hum Pathol* **25:** 797–801

Allan DJ, Harmon BV and Kerr JFR (1987) Cell death in spermatogenesis. In *Perspectives on Mammalian Cell Death,* Pooten CS (ed.), pp. 229–258: Oxford University Press: Oxford

Arai T and Kino I (1995) Role of apoptosis in modulation of the growth of human colorectal tubular and villous adenomas. *J Pathol* **173:** 37–44

Ijiri K and Potten CS (1987) Cell death in cell hierarchies in adult mammalian tissues. In *Perspectives on Mammalian Cell Death,* Pooten CS (ed.), pp. 327–356. Oxford University Press: Oxford

Ikenaga M, Takano Y, Saegusa M, Ohtani Y, Hiki Y, Kakita and Okayasu I (1996) Apoptosis of colon cancers assessed by in situ DNA nick end-labeling method. *Pathol Int* **46:** 33–37

Kaplan EL amd Meier P (1958) Nonparametric estimation from incomplete observations. *J Am Stat Assoc* **53:** 457–481.

Kodama Y, Inokuchi K, Soejima K, Matsusaka T and Okamura T (1983) Growth patterns and prognosis in early gastric carcinoma. *Cancer* **51:** 320–326

Korsmeyer SJ (1992) Bcl-2 initiates a new category of oncogenes: regulators of cell death. *Blood* **80:** 879–886

Krajewski S, Krajewska M, Shabik A, Miyashita T, Wang HG and Reed JC (1994) Immunohistochemical determination of in vivo distribution of bax, a dominant inhibitor of bcl-2. *Am J Pathol* **145:** 1323–1336

Lauwers GY, Scott GV and Karpeh MS (1995) Immunohistochemical evaluation of bcl-2 protein expression in gastric adenocarcinomas. *Cancer* **75:** 2209–2213

Meijerink JPP, Smetsers TFCM, Sloetjes AW, Linders EHP and Mensink EJBM (1995) Bax mutations in cell lines derived from hematological malignancies. *Leukemia* **9:** 1828–1832

Ohbu M, Saegusa M and Okayasu I (1995) Apoptosis and cellular proliferation in oesophageal squamous cell carcinomas: differences between keratinizing and nonkeratinizing types. *Virchows Arch* **427:** 271–276

Oltvai ZN, Milliman CL and Korsmeyer SJ (1993) Bcl-2 heterodimerzes in vivo with a conserved homolog, bax that accelerates programmed cell death. *Cell* **74:** 609–619

Orita H, Matsusaka T, Wakasugi K, Kume K, Fujinaga Y, Fuchigami T and Iwashita A (1992) Clinicopathologic evaluation of recurrence in early gastric cancer. *Jpn J Surg* **22:** 19–23

Pezzala F, Turley H, Kuzu J, Tungekar MF, Dunnill MS, Pierce CB, Harris A, Gatter KG and Mason DY (1993) bcl-2 protein in non-small cell lung carcinoma. *N Engl J Med* **329:** 690–694

Pietenpol JA, Papadopoulos N, Markowitz S, Willson JKV, Kinzler KW, Vogelstein B (1994) Paradoxical inhibition of solid tumor cell growth by bcl-2. *Cancer Res* **54:** 3714–3717

Ranaldi R, Santinelli A, Verdolini R, Rezai B, Mannello B and Bearzi I (1995) Long-term follow-up in early gastric cancer: evaluation of prognostic factors. *J Pathol* **177:** 343–351

Sachs L and Lotem J (1993) Control of programmed cell death in normal and leukemic cells: new implications for therapy. *Blood* **82:** 15–21

Saegusa M, Takano Y, Wakabayashi T and Okayasu I (1995*a*). Apoptosis in gastric carcinomas and its association with cell proliferation and differentiation. *Jpn J Cancer Res* **86:** 743–748

Saegusa M, Takano Y and Okayasu I (1995*b*) Bcl-2 expression and its association with cell kinetics in human gastric carcinomas and intestinal metaplasia. *J Cancer Res Clin Oncol* **121:** 357–363

Saegusa M, Takano Y, Hashimura M, Shoji Y and Okayasu I (1995*c*) The possible role of bcl-2 expression in the progression of tumors of the uterine cervix. *Cancer* **76:** 2297–2303

Saegusa M, Takano Y, Kamata Y and Okayasu I (1996) Bcl-2 expression and allelic loss of the p53 gene in gastric carcinomas. *J Cancer Res Clin Oncol* **122:** 427–432

Shepherd NA, Richman PI and England J (1988) Ki-67 derived proliferative activity in colorectal adenocarcinoma with prognostic correlations. *J Pathol* **155:** 213–219

Shi Y, Sahai BM and Green DR (1989) Cyclosporin A inhibits activation – indeed cell death in T-cell hybridomas and thymocytes. *Nature* **339:** 625–626

Shoji Y, Saegusa M, Takano Y, Ohbu M and Okayasu I (1996) Correlation of apoptosis with tumour cell differentiation, progression, and HPV infection in cervical carcinoma. *J Clin Pathol* **49:** 134–138

Silvestrini R, Veneroni S, Daidone MG, Benini E, Boracchi P, Mezzetti M, Di Fronzo G, Rilke F and Veronesi U (1994) The Bcl-2 protein: a prognostic indicator strongly related to p53 protein in lymph node-negative breast cancer patients. *J Natl Cancer Inst* **86:** 499–504

Sinicrope FA, Ruan SB, Cleary KR, Stephens LC, Lee JJ and Levin B (1995) bcl-2 and p53 oncoprotein expression during colorectal tumorigenesis. *Cancer Res* **55:** 237–241

Smith CA, Williams GT, Kingston R, Jenkinson EJ and Owen JT (1989) Antibodies to CD3/T-cell receptor complex induce death by apoptosis in immature T cells in thymic culture. *Nature* **337:** 181–184

Strater J, Koretz K, Gunthert AR and Moller P (1995) In situ detection of enterocytic apoptosis in normal colonic mucosa and in familial adenomatous polyposis. *Gut* **37:** 819–825

Sugano H, Nakamura K and Kato Y. Pathological studies of human gastric cancer (1982) *Acta Pathol Jpn* **32:** 329–347

Teixeira C, Reed JC, Pratt MAC (1995) Estrogen promotes chemotherapeutic drug resistance by a mechanism involving Bcl-2 proto-oncogene expression in human breast cancer cells. *Cancer Res* **55:** 3902–3907

Tsujimoto Y, Finger LR, Yunis J, Nowell PC and Croce CM (1984) Cloning of the chromosome breakpoint of neoplastic B cells with the t(14;18) chromosome translocation. *Science* **226:** 1097–1099

Walker NI, Bennett RE and Kerr JFR (1989) Cell death by apoptosis during involution of the lactating breast in mice and rats. *Am J Anat* **185:** 19–32

Wyllie AH (1992) Apoptosis and regulation of cell numbers in normal and neoplastic tissues: an overview. *Cancer Metast Rev* **11:** 95–103

© Cancer Research Campaign 1997